# Osteochondral lesions of the talus: Few patients require surgery

**DOI:** 10.1080/17453674.2018.1460777

**Published:** 2018-04-11

**Authors:** Sang Gyo Seo, Jin Soo Kim, Dong-Kyo Seo, You Keun Kim, Sang-Hoon Lee, Ho Seong Lee

**Affiliations:** a Department of Orthopedic Surgery, Asan Medical Center, College of Medicine, University of Ulsan, Seoul;; b Department of Orthopedic Surgery, Gangneung Asan Hospital, College of Medicine, University of Ulsan, Gangneung;; c Department of Radiology, Asan Medical Center, College of Medicine, University of Ulsan, Seoul, Republic of Korea

## Abstract

Background and purpose — The frequency of progression of osteoarthritis and persistence of symptoms in untreated osteochondral lesion of the talus (OCL) is not well known. We report the outcome of a nonoperative treatment for symptomatic OCL.

Patients and methods — This study included 142 patients with OCLs from 2003 to 2013. The patients did not undergo immobilization and had no restrictions of physical activities. The mean follow-up time was 6 (3–10) years. Initial MRI and CT confirmed OCL and showed lesion size, location, and stage of the lesion. Progression of osteoarthritis was evaluated by standing radiographs. In 83 patients, CT was performed at the final follow-up for analyses of the lesion size. We surveyed patients for limitations of sports activity, and Visual Analogue Scales (VAS), AOFAS, and SF-36 were assessed.

Results — No patients had progression of osteoarthritis. The lesion size as determined by CT did not change in 69/83 patients, decreased in 5, and increased in 9. The mean VAS score of the 142 patients decreased from 3.8 to 0.9 (p < 0.001), the mean AOFAS ankle–hindfoot score increased from 86 to 93 (p < 0.001), and the mean SF-36 score increased from 52 to 72 (p < 0.001). Only 9 patients reported limitations of sports activity. The size and location of the lesion did not correlate with any of the outcome scores.

Interpretation — Nonoperative treatment can be considered a good option for patients with OCL.

Osteochondral lesions of the talus (OCL) occur in the articular cartilage and subchondral bone of the talus and are commonly associated with ankle injuries, such as sprains and fractures (Bruns 1997, van Dijk et al. 2010). Treatment of OCL may be operative or nonoperative. With recent advances in arthroscopic techniques, the number of operations for OCL is increasing (van Dijk et al. 1997, Best et al. 2015, Werner et al. 2015).

One indication for operative treatment of OCL is bone fragments within the joint, causing symptoms such as locking. However, other indications remain controversial (Zengerink et al. 2010, Badekas et al. 2013, Hannon et al. 2014). Operative treatment of OCL is costly, and complications such as iatrogenic nerve and articular cartilage injury may occur (Barber et al. 1990, Ferkel et al. 1996, Ferkel et al. 2001, Young et al. 2011, Deng et al. 2012, Vega et al. 2016). In addition, patient satisfaction rates following operative treatment vary between 54% and 89% (Verhagen et al. 2003, Zengerink et al. 2010).

Although several studies have reported on nonoperative treatment of OCL, these were limited by small numbers of patients, short follow-up periods, or inclusion of asymptomatic participants (McCullough and Venugopal 1979, Bauer et al. 1987, Pettine and Morrey 1987, Shearer et al. 2002, Elias et al. 2006, Klammer et al. 2015). In addition, the progress of OCL is still unclear after nonoperative treatment.

In this study, we report the outcome of patients who were diagnosed with symptomatic OCL but were not treated.

## Patients and methods

### Patient demographics

Diagnosis of OCL was initially confirmed for all patients by MRI or CT. Inclusion criteria were as follows: (1) consecutive patients with symptomatic OCL who visited the orthopedic outpatient clinic between 2003 and 2013; (2) patients with a follow-up period of ≥3 years; and (3) patients who had received nonoperative treatment. Patients with advanced osteoarthritis (over Kellgren-Lawrence Grade 2) (Kellgren and Lawrence 1957) at the time of diagnosis and those undergoing surgery during the same period were excluded.

**Figure 1.**
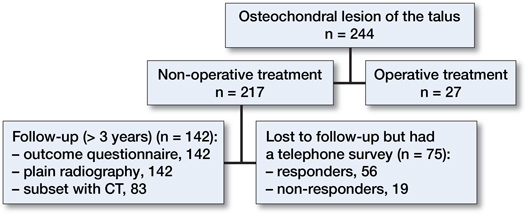
Flowchart of study participants.

244 patients were diagnosed with OCL, among whom 217 (89%) and 27 (11%) received nonoperative and operative treatments, respectively. Among the 27 patients who were surgically treated, 17 had large lesions, severe symptoms, and advanced lesion stage at the time of diagnosis and 10 underwent surgery because of symptomatic deterioration (8 patients) and increased lesion size (2 patients) during the follow-up period. Radiographic follow-up was possible in 142 of 217 patients who received nonoperative treatment (Figure 1).

### Protocol for nonoperative treatment

Patients receiving nonoperative treatment for OCL did not undergo immobilization or restriction of sports activities. From the initial visit, we recommended unlimited daily activity and prescribed the use of NSAIDs as needed for intermittent pain. We explained the natural course of OCL and recommended nonoperative treatment to the patients. Briefly, the nonoperative treatment was composed of “skillful neglect” along with tolerable levels of exercise. Operative treatment was selectively performed in patients whose pain persisted or those who requested surgery for different reasons; some patients wanted to undergo surgery for insurance or military problems.

### Staging and radiographic evaluation

Standing radiographs of the ankle joint (anteroposterior, lateral, and mortise) were evaluated based on the readings of the musculoskeletal radiologist. Osteoarthritis progression (over Kellgren-Lawrence grade 2) (Kellgren and Lawrence 1957) was assessed using plain weight-bearing radiography. The stage of OCL was determined using the Berndt and Harty staging system (Berndt and Harty 1959) with plain weight-bearing radiography and the Ferkel and Sgaglione staging system (Ferkel et al. 1990) with CT. Subchondral cyst and bone-marrow edema were identified using initial MRI. A 9-zone system was used to categorize the location of the lesion, and the lesion size was defined as width, length, and depth.

Follow-up CT was used to assess changes in the lesion and cyst size in 83 patients. CT was paid from research funding, and patients who agree with it participated.

### Clinical evaluation

Baseline data were recorded at the initial assessment, including history of trauma, symptoms related to instability, and limitation of preferred sports activity. Ankle instability, range of motion, and heel alignment were evaluated by physical examination. VAS (Visual Analogue Scales), AOFAS (Kitaoka el al. 1994), and SF-36 scores (Turner-Bowker et al. 2002) were compared between the initial and final follow-up visits.

We investigated the VAS score and limitation of sports activity for the 75 patients lost to follow-up by telephone. 19 did not respond to the telephone survey (Figure 1).

### Statistics

VAS, AOFAS ankle–hindfoot, and SF-36 scores at the initial and final follow-up visits were compared using paired t-tests. The Pearson’s correlation coefficient (r) was used for evaluation of correlations between predisposing factors (sex, age, height, weight, BMI, stage and size of the lesion, and accompanying injury) and the outcome of questionnaires. Interclass correlation coefficients ranged from −1 to +1, with +1 indicating a perfectly positive correlation and −1 indicating a perfectly negative correlation. A value of 0 indicated no correlation.

3 orthopedic surgeons in our author group with 9, 7, and 5 years of experience, respectively, assessed the intraclass correlation coefficient (ICC) of inter-observer reliability for the sizes and stages of the lesions. Prior sample-size estimation and a statistical power analysis indicated the need for assessment of minimum 36 ankle radiographs with CT and MRI to ensure precision in our data. In addition, validation of the differences in lesion size observed with CT and MRI was confirmed by ICC. An ICC value of 1 indicated perfect reliability, and an ICC greater than 0.8 indicated excellent reliability (Donner and Klar 2000). A value of p < 0.05 was considered significant. Statistical analyses were performed using IBM SPSS (Statistics for Windows, Version 21.0; IBM Corp, Armonk, NY, USA).

### Ethics, funding, and potential conflicts of interest

This retrospective study was approved by the Institutional Review Board of Asan Medical Center, which is a tertiary referral hospital. The study has received research support funding from the Asan Institute for Life Sciences, Asan Medical Center, Korea (2016-0634). The authors declared no potential conflicts of interest.

## Results

### Demographics (Table 1)

**Table 1.** Demographic data of the 142 subjects presented as mean values (SD)

Factor	Total
Sex: male/female	82/60
Age (year)	47 (15)
Height (cm)	164 (11)
Weight (kg)	64 (10)
Body mass index	23 (2.8)
Follow-up period (year)	5.7 (2.1)

Of the 217 patients who received nonoperative treatment, 142 patients (82 males) underwent plain weight-bearing radiography at the final follow-up and were evaluated for clinical outcomes and progression of osteoarthritis.

83 (49 males) of these patients also underwent CT at the final follow-up and were evaluated for changes in lesion size.

### Stage and location of lesion at the initial diagnosis

The number of patients with stages I, II, III, and IV lesions was 33, 67, 18, and 24, respectively. Medial lesions were present in 126 patients (89%) and lateral lesions in 16 (11) (Table 2, see Supplemantary data). Initially, the mean width, length, and depth measurements of OCL were 6.9 (1.7–13.1) mm, 9.4 (1.9–19.2) mm, and 5.4 (1.0–15.5) mm, respectively.

### Follow-up radiologic evaluation

The weight-bearing radiographs did not demonstrate progression of ankle osteoarthritis in any of the 142 patients.

**Figure 2.**
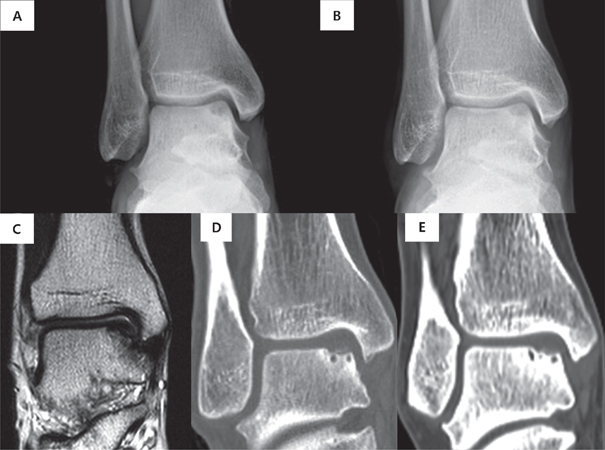
A 29-year-old man with OCL. (A) Initial standing radiograph and (C) initial MRI showed medial OCL. (D) CT at 1-year follow-up, (B) standing radiograph and (E) CT at 9-year follow-up showed no change in lesion size. The AOFAS ankle–hindfoot score improved from 92 to 100.

**Figure 3.**
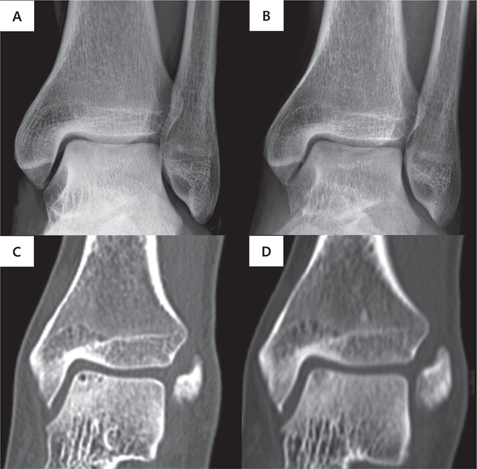
A 53-year-old man with OCL. (A) Initial standing radiograph and (C) CT. (B) Standing radiograph and (D) CT at 7-year follow-up. The lesion size decreased, and the AOFAS ankle–hindfoot score improved from 73 to 100.

**Figure 4.**
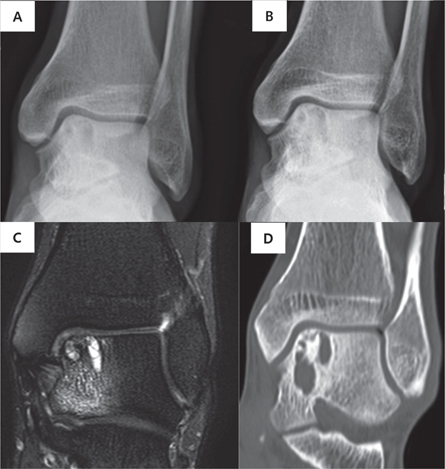
A 48-year-old man with OCL. (A) Initial standing radiograph and (C) MRI. (B) Standing radiograph and (D) CT at 3.8-year follow-up. Although the lesion size increased, the AOFAS ankle–hindfoot score improved from 71 to 80.

**Figure 5.**
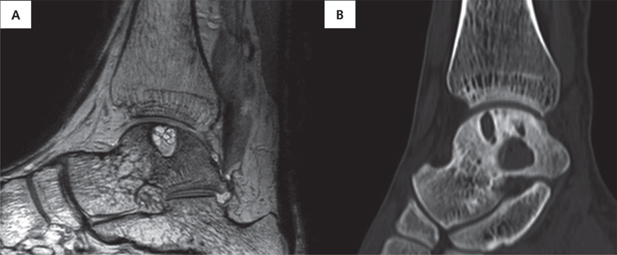
A 59-year-old man with OCL. (A) Initial MRI and (B) CT at 4-year follow-up. Although we recommended surgery to the patient at the first visit, the patient refused the operation because the symptoms were tolerable. The lesion size was increased on the CT at 4-year follow-up. However, the patient did not have worse symptoms and still did not want surgery.

Of the 83 patients who underwent follow-up CT (see Figure 1), 69 patients had no change in lesion size (Figure 2), 5 had decreased lesion size (Figure 3), and 9 had increased lesion size (Figures 4 and 5). Radiographic measurements of the lesion size showed excellent interobserver reliability (ICC, 0.901–0.933).

Initial depth of OCLs was larger (p = 0.02) in the increased-size group compared with that in the group with no change in lesion size, but there was no significant difference in the initial width (p = 0.09) or length (p = 0.1) of OCL between the 2 groups.

In the group with decreased lesion size, there was no significantly difference in the initial width (p = 0.2), length (p = 0.2), or depth (p = 0.6) of OCL in the decreased-size group compared with the no-change group.

All patients in the increased-size group had medially located lesions. Among these, 6 patients were in stage II, 3 in stage I, and none in stage III or IV. Age, sex, and BMI did not correlate with changes in lesion size.

### Clinical evaluation

A history of trauma was detected in 116 of the 142 patients. The most common reported injury was ankle sprain (90 patients), followed by ankle fracture (13 patients) and contusion (11 patients). 23 patients had symptoms of ankle instability.

### Outcome questionnaire

The mean VAS score decreased from 3.8 (1–8) at the initial visit to 0.9 (0–4) at the final follow-up (p < 0.001). The mean AOFAS ankle–hindfoot score improved from 86 (41–93) at the initial visit to 93 (65–100) at the final follow-up (p < 0.001). The mean SF-36 score increased from 52 (30–90) to 71 (37–97) (p < 0.001). Only 9 patients reported limitation of their preferred sports activity.

Even in the increased-size group, the mean VAS score decreased from 4.7 (1–8) at the initial visit to 0.8 at the final follow-up, the mean AOFAS ankle–hindfoot score improved from 82 (65–96) to 93 (71–100), and the mean SF-36 score increased from 55 (36–87) to 77 (52–96).

### Correlation between outcome questionnaire and predisposing factors

In female patients, SF-36 scores at final follow-up were lower than those in male patients (p = 0.01). Moreover, a negative correlation was observed between age and SF-36 scores (p< 0.001). However, there was no significant correlation between AOFAS ankle–hindfoot scores and sex (p = 0.2) or age (p = 0.05). Height, weight, and BMI had no influence on the results of the outcome questionnaires. In addition, the location and size of the lesion at the initial visit did not affect outcome scores. There were also no clinical correlations between outcome scores and associated injury (bone-marrow edema and bone cyst). At the final follow-up, the higher the stage, the higher the VAS (p = 0.02) and the lower the AOFAS ankle–hindfoot scores (p = 0.04) (Table 3, see Supplemantary data).

### Telephone evaluation for radiologic follow-up lost patients

Among 56 of the 75 patients lost to radiological follow-up but who responded to the telephone survey, 50 were still undergoing nonoperative treatment. None of the patients reported limitation of their preferred sports activity, and the mean VAS score was 1.0 (0–3). Among the 56 patients who responded, 6 had undergone operative treatment at another hospital. The mean follow-up period of the telephone survey was 5.0 (3.5–9.4) years.

## Discussion

Numerous studies have reported outcomes for operative treatment of OCL (Kumai et al. 1999, Taranow et al. 1999, Murawski and Kennedy 2013) but few have reported on results of nonoperative treatment and natural history, and the numbers of patients in these studies are low (McCullough and Venugopal 1979, Bauer et al. 1987, Elias et al. 2006). Patient satisfaction with nonoperative treatment was high and the lesions showed little worsening on radiological imaging.

Klammer et al. (2015) observed 48 patients with OCL using only plain radiography and VAS scores for at least 2 years (max x years) and reported that 41 had no symptoms or had a reduction in VAS scores to ≤3.0. Bauer et al. (1987) followed 30 patients with OCL for an average of 21 years and reported that the most of the patients’ symptoms improved, with only 2 patients developing ankle arthrosis and 1 with worsening of symptoms. Other studies on nonoperative treatments for OCL have also reported relatively favorable results (McCullough and Venugopal 1979, Pettine and Morrey 1987, Shearer et al. 2002, Elias et al. 2006).

Because of concerns of possible progression to osteoarthritis or increase in lesion size, many patients diagnosed with OCL are recommended for operative treatment, even when their symptoms are not severe. In fact, among the 142 patients included in this study, 104 had been recommended operative treatment at another hospital or clinic. We found no changes in talar tilt or progression to osteoarthritis in our patients with 10 years of follow-up.

In addition, only 9 of 83 patients who underwent follow-up CT (see Figure 1) showed an increase in lesion size (mean follow-up, 5 years); in the others, lesion size shrank or remained the same, similar to the study by Klammer et al. (2015).

The 9 patients with increased lesion size had a favorable clinical outcome, and it is noteworthy that age, sex, and BMI were not associated with an increase in lesion size. It is also clinically meaningful that the increase in lesion size mainly occurred with large cystic lesions (initial mean depth: 8.3 mm in the increased lesion-size group and 5.4 mm in all patients) but not at stages III and IV. This means that lesions in which bony fragment displacement has already occurred will have little change in size in the future, even at higher OCL stages. Therefore, it is necessary to carefully evaluate changes in stage II cystic lesion size. Although there were no differences in initial symptoms or causes of OCL in the increased-size group, it is necessary to consider the possibility that cystic lesions of increasing size may be in a different category because of the fact that lesions in the increased-size group were limited to stages I and II. This is supported by the fact that degenerated fibrous tissue with a focal osteocartilaginous component was found on histological examination of the patients who underwent surgery because of the increase in lesion size.

Our study included 89% medial and 11% lateral lesions, which is a higher rate of medial lesions than in previous studies. Bernt and Harty (1959) reported a rate of lateral lesions as high as 43%, and Klammer et al. (2015) have reported a 34% rate of lateral lesions. In our study, all patients in the increased-size group had medial lesions. However, it is possible that more patients with medial lesions were included during patient selection because they had mild symptoms, and this should be taken into account when analyzing the results of our study.

Only the stage of the lesion was found to be a factor affecting the outcome of nonoperative treatment. This is similar to previous studies, which also concluded that the higher the stage of the lesion, the longer the duration of symptoms. However, in our study of 142 patients, 42 had lesions that were at stages III and IV and most of these patients had good clinical outcomes. We found no change in the size or progression of osteoarthritis, even at stages III and IV. Therefore, except in cases of intra-articular loose bodies that may cause locking in the ankle joint, we also consider nonoperative treatment to be indicated for patients with stages III and IV lesions.

The 10 patients who underwent surgery during the follow-up period were operated on an average of 8 months after the final follow-up because of aggravation of symptoms (8 patients) without increased lesion size, or increased lesion size (2 patients).

Our study has some limitations. First, there was a selection bias; the study included only patients selected for nonoperative treatment. Therefore, a comparison of outcomes with operative treatment was not made. In addition, because our hospital is a tertiary hospital, there is a selection bias in this regard. Second, our study had a retrospective design. In the future, randomized controlled trials of patients receiving operative and nonoperative treatment for symptomatic OCLs will be helpful to establish clinical practice guidelines. Third, our study was the result of a mid-term follow-up. Long-term follow-up for changes in lesion size and progression of osteoarthritis will be required as well.

In summary, nonoperative treatment of patients with OCL had a satisfactory clinical outcome after a mean follow-up of 6 years. Radiologically, no progression to degenerative osteoarthritis was observed. Nonoperative treatment can be considered a good option for patients with OCL.

### Supplementary data

Tables 2 and 3 are available as supplementary data in the online version of this article, http://dx.doi.org/10.1080/17453674.2018.1460777

SGS developed the methodology, performed the analysis, and wrote the manuscript. JSK and DKS developed the methodology and performed the analysis. YKK collected the data. HSL designed the study, performed the analysis, and wrote the manuscript.

*Acta* thanks Marc G. Romijn and other anonymous reviewers for help with peer review of this study.

## Supplementary Material

IORT_A_1460777_SUPP.pdf
