# Supplementary material for: Osteochondral lesions of the talus: Few patients require surgery
Source: Acta Orthop. 2018 Apr 11;89(4):462–7. doi: 10.1080/17453674.2018.1460777 (PMC6600130; doi:10.1080/17453674.2018.1460777)
Supplement: IORT_A_1460777_SUPP.pdf [file IORT_A_1460777_SM9343.pdf]

Supplementary data

Table 2. Location of osteochondral lesions

Type	Medial	Lateral
Anterior	4	0
Middle	87	9
Posterior	35	7
Total	126	16

There were no central lesions.

Table 3. Correlation between outcome questionnaire and predisposing factors. Data are Pearson correlation coefficients (p-value)

Factor	VAS last	AOFAS ankle-hindfoot scale last	SF-36 last
Gender	–0.074 (0.4)	0.079 (0.2)	–0.188 (0.01)
Age (year)	–0.131 (0.1)	0.134 (0.1)	–0.337 (0.00)
Height (cm)	0.224 (0.1)	–0.088 (0.3)	0.106 (0.2)
Weight (kg)	0.085 (0.5)	–0.061 (0.3)	0.032 (0.4)
BMI	–0.200 (0.1)	0.133 (0.2)	–0.154 (0.1)
Stage	0.193 (0.02)	–0.150 (0.04)	–0.015 (0.4)
Bone marrow edema	–0.038 (0.7)	0.082 (0.2)	–0.092 (0.2)
Bone cyst	0.002 (1.0)	–0.041 (0.3)	0.013 (0.4)
Location	0.134 (0.1)	–0.001 (0.5)	–0.076 (0.2)
Size			
Width (mm)	–0.016 (0.4)	0.046 (0.3)	0.119 (0.1)
Length (mm)	0.061 (0.2)	–0.027 (0.4)	0.032 (0.4)
Depth (mm)	0.032 (0.4)	–0.025 (0.4)	0.024 (0.4)
Total	0.014 (0.4)	0.010 (0.5)	0.019 (0.4)
